# Notes and News

**Published:** 1891-05-30

**Authors:** 


### Notes and News.

Collected and Communicated.

On Sunday last the Jewish Convalescent Home at Hove, which is connected with that at Norwood, was formally opened. The Home will not, however, be ready for the reception of inmates for another fortnight. It has only recently been erected and has already cost nearly £3,000. The building is of red brick with glazed tiles, and is erected in Elizabethan style. It will accommodate twenty-two patients, and is open to both sexes, the house being divided into two sections, and from its capital position in a popular locality should have a very prosperous career. Mr. A. E. Sassoon presided over the opening ceremony which began with a short service, conducted by Dr. Adler. Mr. Sassoon, in the course of his address, spoke of the great need of such an institution, and appealed for liberal support. The proceedings terminated with the singing of the National Anthem.

The Queen received an enthusiastic greeting from the loyal people of Derby when she arrived on Thursday, the 21st inst., to lay the foundation stone of the new Infirmary. The streets and buildings were profusely and tastefully decorated, and Her Majesty's progress through the town really resolved itself into a Royal pageant. On reaching the marquee where the ceremony was to take place, the Royal visitors were received by the High Sheriff, the Mayor, and Lord Scarsdale, who represented Sir William Evans, and read an address of welcome. Her Majesty responded, saying that she earnestly hoped that this undertaking, in which she took a deep personal interest, might effectually contribute to the relief of human suffering, and to the health of those whose career of honourable labour had been interrupted by accident or sickness. The formal laying of the stone was then carried out, the Bishop of Southwell offering special prayers for the occasion.

The annual meeting of the subscribers to the Devonshire Hospital and Buxton Bath Charity was held on May 2, Dr. Robertson, M.D., F.R.C.P., presiding. The report and annual statement of accounts were taken as read, and the Chairman then delivered an addres to the subscribers in which he explained the great structural and sanitary advantages of the hospital, which enabled them to give their patients such unrivalled benefits. The hospital fully maintains its position of extensive usefulness to the many sufferers from diseases of a rheumatic or gouty character. Since May 1, 1890, 2,363 patients have been admitted, a large proportion of whom had been discharged as improved. This hospital occupies a great position among the charitable institutions of the realm and has the support of a very large list of annual and life subscribers, among whom are an important number of Poor Law Unions and Friendly Societies. After a vote of thanks to the Chairman, some formal business was gone through and the meeting ended.

At the dinner in aid of the funds of the North-Eastern Hospital for Children, held on the 15th inst. in the Venetian Chamber of the Holborn Restaurant under the presidency of the Duke of Connaught, half the sum appealed for (£4,000) was subscribed in the room. This appeal had been necessitated by the failure of a portion of the income (legacies and special gifts), and also by the drainage of the hospital having to be overhauled. A large number of ladies attended the dinner, among whom were Lady Tweeddale, Lady Yarborough, Lady F. J. Fitzroy, Lady Leucha Warner, Lady Morell Mackenzie, Mrs. W. A. T. Amherst.

The House Committee of the Carmarthenshire Infirmary state in their forty-fourth annual report that the efficiency and condition of the hospital are most satisfactory. The Committee call attention to the statement of accounts which shows an adverse balance due to the Treasurer of £13 12s. 7d. The adverse balance last year was £69 15s. 8d., so that the pecuniary position of the institution is much more favourable now than then. This is mainly due to an increase in donations, as there is a decrease in the subscriptions as compared with those for last year. A sub-committee that was appointed early in January, 1891, to enquire into the financial condition and management of the infirmary, after a long and careful investigation, came to the conclusion that retrenchment in no department was possible without either lessening the efficiency of the institution or reducing the number of in-patients.

The 13th annual report of the Home Hospitals Association gives every detail of the work throughout the year, and has attached to it a list of the subscriptions of the Life Governors since its foundation. During 1890, there were 463 applicants for admission, of whom 324 were admitted, an increase of 23 over the admissions for 1889. The receipts are, however, rather less than in the previous year, the cheaper rooms having been more frequently occupied than in former years. The Nurses' Home, which was opened in December, 1888, has proved very successful. There are now 12 nurses on the staff which number will shortly be increased to 16. The Committee have taken the house, 18, Fitzroy Square, on a short lease, have suitably furnished it, the whole cost being defrayed out of the money realised by the Home, none of the hospital money being devoted to that purpose. The income for the year from the Home was £893, and the expenditure £938, but as the year began with a balance of £87, there was still £42 in hand at the close of the year.

The Public Health Committee of Leith are seriously considering the necessity of providing a suitable hospital for the borough. Some action on their part is rendered absolutely necessary by the want of proper accommodation at Leith Hospital for the treatment of infectious cases and by the resolution of the directors of that institution not to receive such patients after October next. This decision of the directors, which they came to without consulting the subscribers or the public, has caused some indignation in the town. Leith Hospital, from its very constitution and character, is admittedly a public hospital, and for the last two years the local authority has contributed, under agreement with the directors, an annual sum of from £1,000 to £1,200 to its support. This is a very large sum, but the directors, while insisting on their full payment out of the local rates in terms of the agreement, failed to provide suitable accommodation and safe and proper treatment for the patients admitted. Such a state of things could not continue, and the public look to the local authority to take prompt action in the matter.

